# 

**Published:** 1894-02

**Authors:** 


### CONTENTS FOR FEBRUARY, 1894.

ORIGINAL COMMUNICATIONS. PAGE.

Observations on the Results of Removal of Diseased Uterine Appendages. By M. A. CROCKETT, A. B., M. D. .... 385

Prostatic Diseases, with Special Reference to the Prostate of Old Age. By MARCELL HARTWIG, M. D. .... 397

CLINICAL REPORT.

Clinical Memoranda from the Surgical Clinic at the Sisters' of Charity Hospital. By HERMAN MYNTER, M. D. .... 415

SOCIETY PROCEEDINGS.

Medical Society of the County of Erie .... 420

CORRESPONDENCE.

Letter from Mr. LAWSON TAIT .... 424

EDITORIAL.

Symphyseotomy .... 430
Topics of the Month .... 433

Personal .... 435
Obituary .... 436
College Notes .... 437
Society Meetings .... 438
Book Reviews .... 442
Literary Notes .... 446
Miscellany .... 448

